# Variation in HIV-1 Nef function within and among viral subtypes reveals genetically separable antagonism of SERINC3 and SERINC5

**DOI:** 10.1371/journal.ppat.1008813

**Published:** 2020-09-14

**Authors:** Steven W. Jin, Francis M. Mwimanzi, Jaclyn K. Mann, Mwebesa Bosco Bwana, Guinevere Q. Lee, Chanson J. Brumme, Peter W. Hunt, Jeff N. Martin, David R. Bangsberg, Thumbi Ndung’u, Zabrina L. Brumme, Mark A. Brockman

**Affiliations:** 1 Faculty of Health Sciences, Simon Fraser University, Burnaby, Canada; 2 HIV Pathogenesis Programme, The Doris Duke Medical Research Institute, University of KwaZulu-Natal, Durban, South Africa; 3 Faculty of Medicine, Mbarara University of Science and Technology, Mbarara, Uganda; 4 British Columbia Centre for Excellence in HIV/AIDS, Vancouver, Canada; 5 Department of Medicine, University of British Columbia, Vancouver, Canada; 6 School of Medicine, University of California, San Francisco, United States of America; 7 School of Public Health, Oregon Health Science University, Portland, United States of America; 8 Africa Health Research Institute, Durban, South Africa; 9 Ragon Institute of MGH, MIT, and Harvard University, Cambridge, United States of America; 10 Max Planck Institute for Infection Biology, Berlin, Germany; 11 Division of Infection and Immunity, University College London, London, United Kingdom; 12 Department of Molecular Biology and Biochemistry, Simon Fraser University, Burnaby, Canada; Miller School of Medicine, UNITED STATES

## Abstract

HIV Nef counteracts cellular host restriction factors SERINC3 and SERINC5, but our understanding of how naturally occurring global Nef sequence diversity impacts these activities is limited. Here, we quantify SERINC3 and SERINC5 internalization function for 339 Nef clones, representing the major pandemic HIV-1 group M subtypes A, B, C and D. We describe distinct subtype-associated hierarchies for Nef-mediated internalization of SERINC5, for which subtype B clones display the highest activities on average, and of SERINC3, for which subtype B clones display the lowest activities on average. We further identify Nef polymorphisms that modulate its ability to counteract SERINC proteins, including substitutions in the N-terminal domain that selectively impair SERINC3 internalization. Our findings demonstrate that the SERINC antagonism activities of HIV Nef differ markedly among major viral subtypes and between individual isolates within a subtype, suggesting that variation in these functions may contribute to global differences in viral pathogenesis.

## Author summary

Cellular restriction factors dampen viral replication and reduce pathogenesis. In response, HIV has evolved a variety of mechanisms to counteract restriction factors, including the ability of its Nef protein to internalize Serine incorporator (SERINC) proteins 3 and 5 from the infected cell surface, thereby enhancing infectivity of viral particles. Nef displays substantial sequence diversity, but how this impacts SERINC antagonism remains unclear. To examine this, we used cell culture models to measure SERINC internalization function for 339 participant-derived Nef clones, representing four globally relevant HIV-1 group M subtypes. We observed significant variability in Nef function among circulating viral strains and subtypes. We also identified naturally occurring Nef mutations that modulate its ability to counteract SERINC proteins, including some that selectively impair SERINC3 internalization. These findings uncover viral features that may contribute to global differences in HIV pathogenesis and provide new insight into the interactions between Nef and SERINC restriction factors that can inform future mechanistic studies.

## Introduction

Cellular restriction factors impede HIV replication [1–5]. In response, HIV has evolved strategies to counteract these intrinsic host antiviral molecules [6]. Among the most recent restriction factors to be identified are members of the Serine incorporator (SERINC) family of multi-pass transmembrane proteins, namely SERINC3 and SERINC5, which are antagonized by the HIV accessory protein Nef [7–9]. Nef is a 27–35 kD cytosolic protein that plays a crucial role in viral pathogenesis [10–12]. Through its various interactions with cellular protein trafficking machinery, Nef internalizes CD4 [13], HLA class I [14–16], SERINC3 and SERINC5 [7, 8] as well as other proteins from the surface of infected cells, thereby enhancing viral replication and evading host immune responses. The presence of SERINC proteins in viral particles interferes with the generation of a fusion pore following binding to a target cell [17], thus impeding viral entry. SERINC5 appears to be more potent at blocking HIV entry compared to SERINC3 [8]. As a result, most reports focus on Nef’s ability to antagonize SERINC5, and its impact on SERINC3 remains relatively understudied. While the mechanism of SERINC’s antiviral activity has not been fully clarified, a recent study found that SERINC5 disrupts viral Envelope (Env) clusters on the virion surface through an indirect process [18]; however, other studies suggest that a direct interaction between Env and SERINC5 may result in conformational changes to Env that impair its function [19, 20]. Regardless of the precise mechanism(s) of action, by removing SERINC proteins from the infected cell surface, Nef reduces their incorporation into virions and counteracts their antiviral function [7, 8].

The HIV-1 group M (Main) pandemic strains comprise nine genetically diverse subtypes (A–D, F–H, J and K) and nearly 100 circulating recombinant forms [21]. Viral subtype is a key determinant of clinical progression [22, 23], as demonstrated by the observation that subtypes A and C are associated with reduced pathogenesis compared to subtype D in regions where these viruses co-circulate [24–26]. While strain-specific differences in simian immunodeficiency virus (SIV) Nef-mediated SERINC5 antagonism correlate with the prevalence of lentiviral infections in wild primates [27], the impact of global HIV Nef sequence diversity on its SERINC antagonism activities remains incompletely characterized. Consistent with the hypothesis that naturally occurring variation in Nef function may play an analogous role in HIV infection, initial studies reported that Nef clones from infected individuals differed in their ability to antagonize SERINC proteins [7, 27]. Prior work from our group also indicates that naturally occurring variation in Nef sequences yields substantial diversity in several of Nef’s best-characterized functions, including CD4 and HLA class I internalization [28–32]. Moreover, we recently reported that Nef clones isolated from HIV elite controllers, who suppress plasma viremia in the absence of antiretroviral therapy, often display reduced ability to internalize SERINC5 [33], suggesting that variability in Nef-mediated SERINC antagonism contributes to clinical outcome in at least some individuals.

To date, no large-scale studies have characterized the extent to which Nef’s ability to antagonize SERINC proteins varies among global HIV subtypes. Here, we examined a diverse panel of 339 Nef clones isolated from individuals infected with subtypes A, B, C, or D for their ability to internalize SERINC3 and SERINC5. We observed that SERINC3 and SERINC5 antagonism activity varies markedly among circulating Nef isolates and between viral subtypes. Notably, while subtype B Nef clones displayed superior abilities to internalize SERINC5, they were frequently impaired in their ability to internalize SERINC3. We further identified natural polymorphisms in Nef that were associated with variation in SERINC3 and/or SERINC5 internalization function, including substitutions at Nef residues 8 and 11 that impaired its ability to counteract SERINC3, but not SERINC5. Our results indicate that variation in Nef-mediated SERINC antagonism may contribute to differences in pathogenesis among HIV subtypes.

## Results

### SERINC internalization function varies markedly among HIV Nef isolates

**Fig 1:**
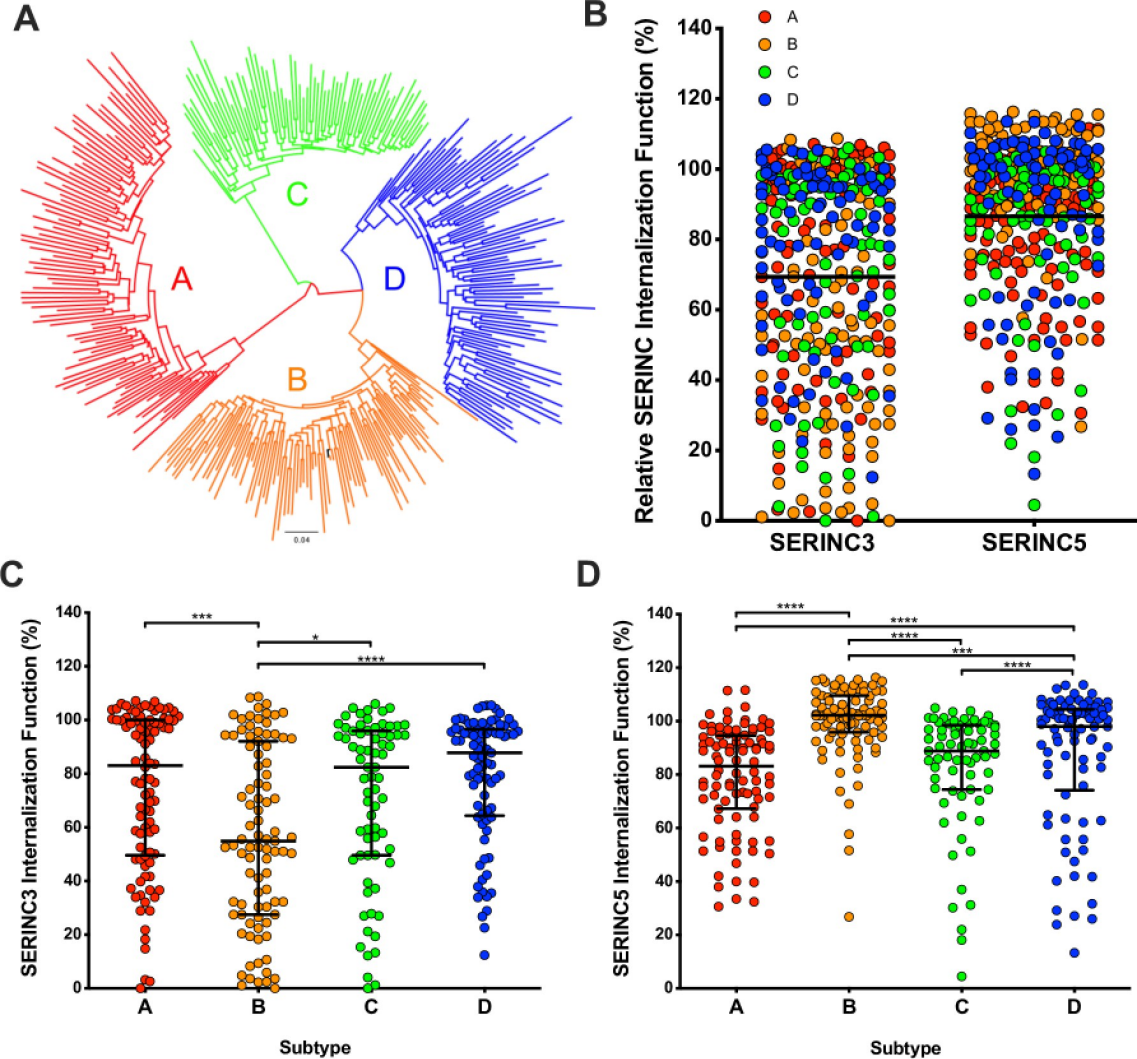
Nef-mediated SERINC3 and SERINC5 internalization function varies among circulating Nef alleles representing globally relevant subtypes. **(A)** A maximum likelihood phylogenetic tree of 339 participant-derived HIV Nef clones is shown. Subtype A clones are represented in red, subtype B in orange, subtype C in green and subtype D in blue. The subtype B NL4.3 Nef strain (black) was included as a reference. **(B)** The relative ability of all Nef clones to internalize SERINC3 (left) or SERINC5 (right) is shown. SERINC internalization was assessed by flow cytometry following co-transfection of CEM T-cells with Nef and SERINC(iHA) (also see **S1 Fig)**, and values were normalized to that of the NL4.3 Nef control (set as 100%). **(C)** The relative ability of each Nef clone to internalize SERINC3 is shown, stratified by subtype. **(D)** The relative ability of each Nef clone to internalize SERINC5 is shown, stratified by subtype. Bars represent the median and the interquartile ranges. Statistical analysis was performed using the Mann-Whitney U-test and significant results are indicated by asterisks (* = p<0.05; ** = p≤0.01; *** = p≤0.001; **** = ≤0.0001).

We assembled a diverse panel of 339 HIV Nef clones consisting of unique isolates obtained from individuals residing in Canada, Uganda or South Africa who were infected with HIV subtype A, B, C, or D, as reported previously [30]. These *nef* sequences clustered into distinctive subtypes (Fig 1A) and did not include viral recombinants that could confound analysis. Each Nef clone was characterized for its ability to internalize SERINC3 and SERINC5 from the cell surface using a transient expression assay (described in the Methods). Briefly, CEM CD4 T cells were co-transfected with plasmids expressing a Nef clone and one of the SERINC proteins, which was modified to encode an internal HA tag (iHA). SERINC expression was quantified by detecting the HA tag on the surface of non-permeabilized cells using flow cytometry. Consistent with prior studies [8, 33], a Nef clone derived from the subtype B SF2 strain efficiently internalized SERINC5 but not SERINC3, while a Nef clone derived from the subtype B NL4.3 strain internalized both proteins (S1 Fig). As such, SERINC internalization results for each Nef clone were normalized to those of NL4.3 Nef, such that activities greater than or less than NL4.3 Nef are indicated by values above or below 100%, respectively.

The 339 Nef clones differed widely in their ability to internalize the SERINC proteins: the median SERINC3 internalization function of all clones relative to the NL4.3 Nef control was 78 [interquartile range (IQR) 48–97]%, while the median SERINC5 internalization function was 94 [IQR 77–102]% (Mann-Whitney; p<0.0001) (Fig 1B). The observation that SERINC5 internalization was overall more conserved than SERINC3 internalization suggests that it may be less crucial for Nef to maintain the latter activity *in vivo*, in at least some cases. This result is also consistent with prior *in vitro* studies demonstrating that SERINC3 displays lower antiviral activity compared to SERINC5 [8]. Stratification of the data by viral subtype revealed significant differences in Nef-mediated SERINC3 and SERINC5 internalization function as well as distinct functional hierarchies for the two proteins (Fig 1C and 1D). Specifically, subtype B Nef clones displayed the highest function for SERINC5 internalization (median 102 [IQR 96–110] %) followed closely by subtype D clones (median 98 [IQR 74–104] %) (Mann-Whitney; p = 0.0005), while clones derived from subtype C (median 89 [IQR 74–99] %) and subtype A (median 83 [IQR 67–95] %) displayed lower abilities to internalize this protein (pairwise Mann-Whitney; all p<0.005 vs. B and D) (Fig 1D). In contrast, SERINC3 internalization function was comparable among Nef clones derived from subtypes D (median 88 [IQR 64–97]%), A (median 83 [IQR 50–100]%), and C (median 82 [IQR 50–96]%) (pairwise Mann-Whitney; all p>0.05), whereas subtype B clones displayed lower ability to internalize this protein (median 55 [IQR 28–92]%) (pairwise Mann-Whitney; all p<0.05).

Consistent with the relatively high functional variability observed among Nef clones, we found only a weak association between SERINC3 and SERINC5 internalization functions when all clones were considered (Spearman R = 0.21; p<0.0001) (S2 Fig); however, this improved notably when subtype B clones were excluded (R = 0.47; p<0.0001). Furthermore, stratification of these data according to viral subtype resulted in moderate to strong correlations between SERINC3 and SERINC5 internalization function for Nef clones from subtypes A, C or D (R = 0.37 to 0.62; all p<0.001), but there was no similar association for subtype B clones (R = 0.04; p = 0.74). Next, we assessed possible associations between SERINC antagonism and Nef’s other major internalization functions, namely downregulation of CD4 and HLA class I (HLA-I), which we reported previously for this panel of Nef clones [30] (S3 Fig). Consistent with our study of Nef clones derived from HIV controllers [33], a strong correlation was observed between SERINC5 and CD4 internalization functions (Spearman R = 0.53; p<0.0001), while a more moderate association was found between SERINC5 and HLA class I internalization (R = 0.32; p<0.0001). Overall, SERINC3 internalization function correlated modestly with CD4 downregulation (R = 0.20; p<0.0001), but this association was strengthened when subtype B clones were excluded (R = 0.41; p<0.001). A modest inverse association was found between SERINC3 and HLA-I internalization functions (R = -0.19; p<0.001), but this was not apparent after removal of subtype B clones and thus its relevance is unclear. Finally, to assess whether variable Nef expression or stability could be responsible for the observed differences in SERINC antagonism function, we quantified steady-state protein levels for 61 randomly selected Nef clones (representing all subtypes) by Western blot. We observed no association between protein detection and function for these clones.

Together, these results demonstrate substantial variability in SERINC3 and SERINC5 internalization function among natural HIV Nef isolates and also identify a more pronounced impairment in SERINC3 internalization among subtype B clones. Stronger associations between SERINC and CD4 downregulation are consistent with other data suggesting a shared cellular mechanism of Nef-mediated internalization for these proteins [7, 9].

### Nef-mediated antagonism of SERINC3 and SERINC5 enhances HIV infectivity

**Fig 2:**
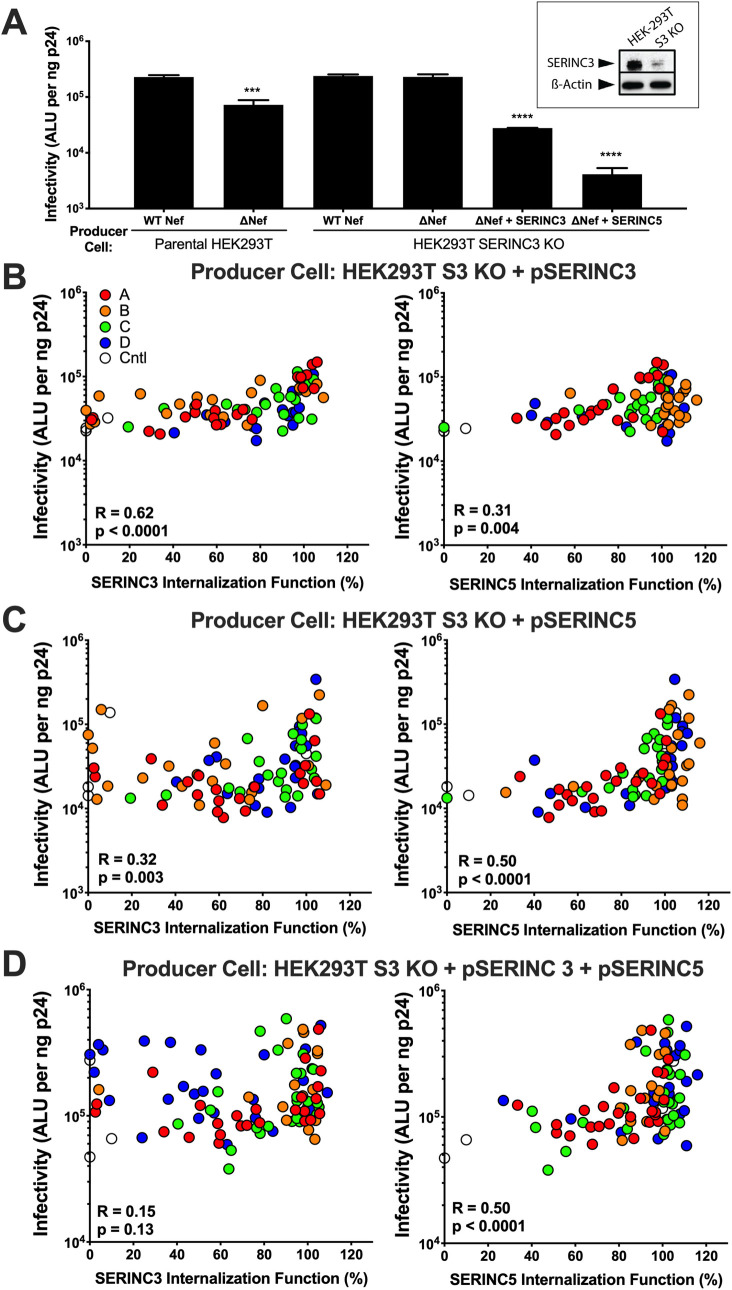
Internalization of SERINC3 and SERINC5 correlates with viral infectivity enhancement. **(A)** The relative infectivity of NL4.3 or Nef-deleted (ΔNef) viruses produced in absence or presence of SERINC proteins is shown. HIV producer cells (either parental HEK293T cells or SERINC3 KO cells) were co-transfected with viral plasmid plus empty vector or SERINC(iHA) plasmids. Results are reported as absolute light units (ALU; log10) based on luminescence from TZM-bl reporter cells following infection with a standardized amount of viral stock (normalized to ng p24). Bars represent the mean and standard deviation based on three independent experiments. Statistical analyses were conducted using the unpaired Students T test. Significant differences compared to Nef controls are indicated by asterisks (*** = p≤0.001; **** = ≤0.0001). Inset: CRISPR/Cas9-mediated knockout of SERINC3 in a HEK293T cells was validated using Western blot. SERINC3 was detected using rabbit polyclonal antibody; cellular β-actin was used as a loading control. **(B)** Correlations between SERINC3 (left) or SERINC5 (right) internalization activity (x-axis) and viral infectivity (y-axis; log10) are shown for ΔNef viruses produced in SERINC3 KO cells co-transfected with selected Nef clones and SERINC3(iHA). A total of 82 Nef clones were examined (19–22 per subtype). **(C)** Correlations between relative SERINC3 (left) or SERINC5 (right) internalization activity (x-axis) and viral infectivity (y-axis; log10) are shown for ΔNef viruses produced in SERINC3 KO cells co-transfected with selected Nef clones and SERINC5(iHA). **(D)** Correlations between relative SERINC3 (left) and SERINC5 (right) internalization function (x-axis) and viral infectivity (y-axis; log10) are shown for ΔNef viruses produced in SERINC3 KO cells co-transfected with selected Nef clones plus equal amounts of SERINC3(iHA) and SERINC5(iHA). Statistical analyses were conducted using the Spearman rank correlation test.

To complement the results of SERINC internalization assays, we next investigated the impact of different *nef* alleles on viral infectivity. Such assays have typically produced viruses using HEK293T cells, however, these cells express high levels of SERINC3 relative to CD4 T-cell lines [8], which limits our ability to modulate SERINC3 expression [34]. To overcome this, we disrupted *SERINC3* in 293T cells using CRISPR/Cas9 as described in Methods and validated the SERINC3 knockout (KO) cell line by confirming the infectivity of wild type and Nef-deleted NL4.3 strains produced using these cells (Fig 2A). While Nef enhanced viral infectivity ~three-fold in parental 293T cells [7, 35], the infectivity of wild type and Nef-deleted NL4.3 viruses did not differ when they were produced using SERINC3 KO cells, consistent with the inactivation of this key restriction factor. Furthermore, rescue of SERINC expression in these cells by co-transfection of pSERINC3(iHA) or pSERINC5(iHA) reduced the infectivity of Nef-deleted virus by 8-fold or 55-fold, respectively, confirming that SERINC5 is a more potent restriction factor in this model.

Using the SERINC3 KO cell line, we generated 82 NL4.3-derived viruses encoding selected *nef* alleles in the presence or absence of each SERINC protein. As expected, the infectivity of viruses produced in the presence of SERINC3 alone correlated strongly with our measures of Nef-mediated internalization of this protein (Spearman R = 0.62, p<0.0001) (Fig 2B), but less so with our measures of SERINC5 internalization (Spearman R = 0.31, p = 0.004). Conversely, the infectivity of viruses produced in the presence of SERINC5 alone correlated strongly with our measures of Nef-mediated internalization of this protein (Spearman R = 0.63, p<0.0001), but less so with our measures of SERINC3 internalization (Spearman R = 0.32, p = 0.003) (Fig 2C). The specificity of these correlations supports the notion that Nef uses somewhat distinct mechanisms to internalize SERINC3 and SERINC5.

Notably, when viruses were produced in the presence of both SERINC proteins, infectivity correlated with Nef-mediated SERINC5 internalization (Spearman R = 0.50, p<0.0001), but not with SERINC3 internalization (Spearman R = 0.15, p = 0.13) (Fig 2D). This result indicates that SERINC5 plays a dominant role in modulating HIV infectivity, and is consistent with reports identifying it as the more potent restriction factor [7, 8]. Overall, our data demonstrate that Nef-mediated SERINC internalization function is closely linked with its overall impact on viral infectivity in the presence of SERINC proteins, though other Nef functions may also contribute to this outcome [36].

### Nef polymorphisms associate with SERINC antagonism activity

**Fig 3:**
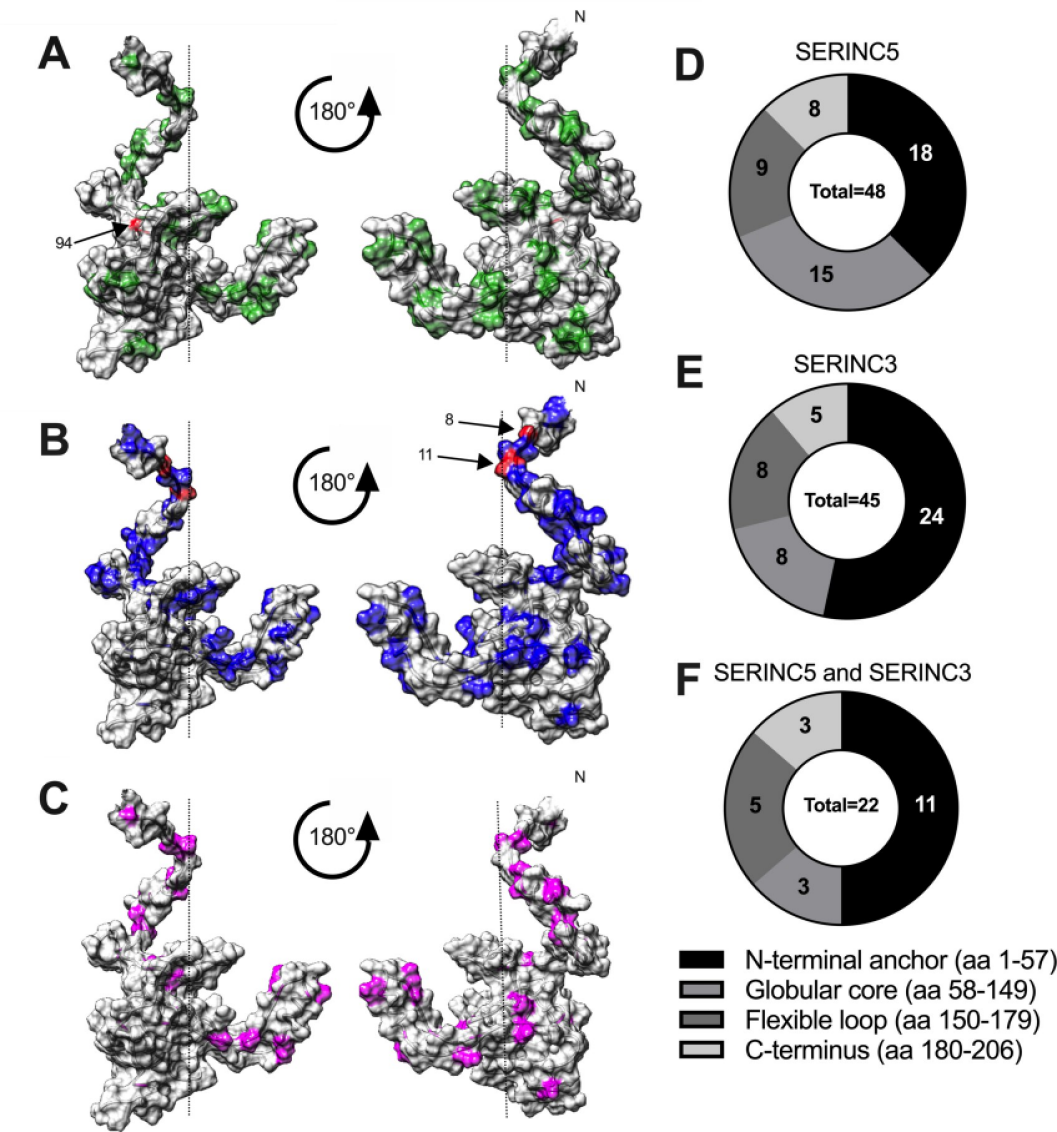
Structural distribution of Nef residues associated with SERINC antagonism. **(A, B, C)** The location of Nef polymorphisms associated with internalization of SERINC5 **(A**, green**)**, SERINC3 **(B**, blue**)**, or both proteins **(C**, magenta**)** are indicated on a composite structural model of HIV Nef (based on PDB 2NEF and 1QA5) (Lamers, PLoS One 2011). Residue 94 (SERINC5, panel **A**) and residues 8 and 11 (SERINC3, panel **B**) are highlighted in red. **(D, E, F)** The distribution of natural polymorphisms associated with internalization of SERINC5 (n = 48) **(D)**, SERINC3 (n = 45) **(E)** or both proteins (n = 22) **(F)** among Nef’s major functional domains is illustrated.

Given the marked variation in SERINC antagonism activity among Nef clones and the substantial differences in the ability of subtype B clones to counteract SERINC5 versus SERINC3, we analyzed our sequence/function dataset in a subtype-specific manner to identify viral polymorphisms associated with each Nef function (see Methods). We identified 73 *nef* polymorphisms, located at 48 Nef residues, associated with SERINC5 internalization function (Mann-Whitney; all p<0.05) (Table 1 and S1 Table). These residues were distributed relatively evenly across Nef’s major domains (i.e. N-terminal anchor, globular core, flexible loop and C-terminal tail) [37, 38] (Fig 3A and 3D), suggesting that natural variation in multiple regions of the protein contribute to this activity. The strongest of these associations occurred at Nef residue 94 in subtype B, where the presence of consensus Lysine correlated with better SERINC5 internalization (Mann-Whitney; p<0.0002). Notably, Nef K94E was associated with reduced SERINC5 internalization function in an independent panel of subtype B Nef clones isolated from HIV elite controllers and progressors published by our group [33]. In that report, we confirmed that K94E impaired Nef’s SERINC5 internalization function and reduced its ability to enhance viral infectivity in the presence of SERINC5. New associations with Nef function that were identified through this analysis, including those at residues 60 and 180 in subtype A, 61 and 197 in subtype B, 88 and 98 in subtype C, and 57 and 114 in subtype D (Mann-Whitney; all p<0.01), warrant further investigation.

**Table 1:** Selected Nef polymorphisms associated with SERINC5 internalization function.

Subtype	Residue	Domain^a^	Amino Acid	Median Function (With)	Median Function (Without)	N (With)	N (Without)	p-value
A	60	Globular core	E	46	85	4	88	0.008
A	60	Globular core	A	85	51	87	5	0.03
A	180	C-terminal	T	76	90	54	38	0.005
A	180	C-terminal	V	90	76	38	54	0.005
B	61	Globular core	Q	109	90	86	5	0.009
B	61	Globular core	Y	82	108	3	88	0.01
B	94	Globular core	K	110	96	77	14	0.0001
B	94	Globular core	E	82	109	7	84	0.006
B	197	C-terminal	E	108	76	88	3	0.006
C	3	N-terminal anchor	G	94	75	48	23	0.009
C	3	N-terminal anchor	N	62	91	6	65	0.02
C	32	N-terminal anchor	A	87	101	64	7	0.005
C	32	N-terminal anchor	T	101	88	3	68	0.02
C	88	Globular core	G	70	91	15	56	0.008
C	88	Globular core	S	91	70	56	15	0.008
C	98	Globular core	D	82	95	34	37	0.006
C	98	Globular core	E	95	82	37	34	0.006
D	57	N-terminal anchor	W	101	51	81	4	0.008
D	114	Globular core	V	101	64	73	12	0.003
D	114	Globular core	I	76	101	10	75	0.02

a Domain nomenclature based on [37, 38].

We also identified 108 *nef* polymorphisms, located at 45 Nef residues, associated with SERINC3 internalization function (Mann-Whitney; all p<0.05) (Table 2 and S2 Table). Of note, these were modestly enriched (24, or 53%) at sites located in the N-terminal anchor (codons 1–57) (Fig 3B and 3E), suggesting that Nef’s ability to internalize SERINC3 may be particularly sensitive to changes in this region. Remarkably, in all subtypes tested, the strongest correlations were located at Nef residues 8 and/or 11, where the presence of consensus Serine or Valine, respectively, correlated with better SERINC3 internalization function. These associations were most statistically significant for clones derived from subtypes A and B (all p<0.00001).

**Table 2:** Selected Nef polymorphisms associated with SERINC3 internalization function.

Subtype	Residue	Domain ^a^	Amino Acid	Median Function (With)	Median Function (Without)	N (With)	N (Without)	p-value
A	8	N-terminal anchor	S	96	40	73	17	4E-06
A	8	N-terminal anchor	R	42	93	9	81	0.002
A	10	N-terminal anchor	I	97	48	65	23	9E-06
A	10	N-terminal anchor	K	36	94	10	78	0.0001
A	11	N-terminal anchor	V	96	37	72	16	1.2E-06
A	11	N-terminal anchor	A	31	92	6	82	0.0003
A	14	N-terminal anchor	P	92	41	82	10	0.002
B	8	N-terminal anchor	S	92	51	37	46	6E-06
B	8	N-terminal anchor	R	51	76	28	55	0.004
B	11	N-terminal anchor	V	94	43	30	47	9E-07
B	11	N-terminal anchor	S	36	69	7	70	0.01
B	14	N-terminal anchor	P	71	39	57	34	0.0009
B	14	N-terminal anchor	A	31	56	8	83	0.04
C	8	N-terminal anchor	R	47	88	5	63	0.02
C	11	N-terminal anchor	V	91	48	52	14	0.001
C	11	N-terminal anchor	A	50	88	4	62	0.04
C	40	N-terminal anchor	Y	93	67	31	40	0.006
C	49	N-terminal anchor	A	66	94	40	31	0.005
C	49	N-terminal anchor	P	96	76	11	60	0.01
D	8	N-terminal anchor	S	94	67	69	13	0.0002
D	8	N-terminal anchor	R	70	93	10	72	0.007
D	14	N-terminal anchor	P	90	38	80	5	0.003
D	14	N-terminal anchor	S	37	89	4	81	0.007
D	40	N-terminal anchor	Y	103	86	4	81	0.007
D	64	Globular core	E	83	95	64	21	0.006
D	100	Globular core	I	104	86	4	81	0.003

a Domain nomenclature based on (37, 38).

In total, 71 Nef residues (of 206, 34%) were associated with either SERINC3 or SERINC5 internalization function. Twenty-two Nef residues were associated with both functions (Fig 3C and 3F; S3 Table), suggesting shared structural motifs or interacting domains that contribute to both activities, while 49 were associated with only one function, suggesting regions of the protein that play a unique role in Nef’s ability to counteract SERINC3 or SERINC5. Notably, Nef residue 94 was not associated with SERINC3 internalization function, and neither residue 8 nor 11 were associated with SERINC5 internalization function, indicating that these two activities are genetically separable.

### Polymorphisms at Nef codons 8 and 11 selectively impair SERINC3 antagonism activity

**Fig 4:**
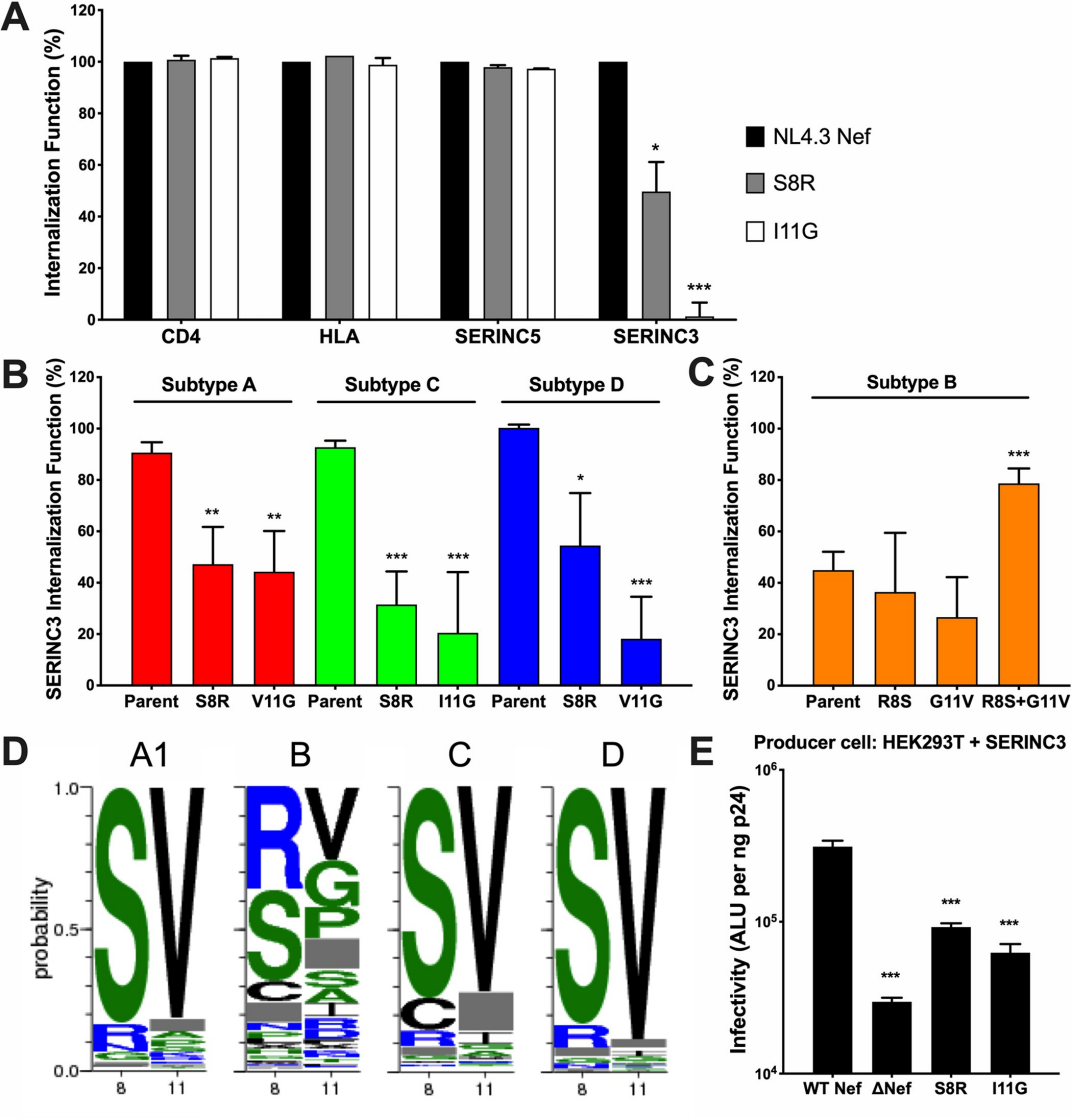
Polymorphisms at Nef residues 8 and 11 selectively impair SERINC3 internalization function. **(A)** The relative abilities of NL4.3 Nef mutants S8R and I11G to internalize CD4, HLA class I, SERINC5 and SERINC3 are shown. Results are normalized to wild type NL4.3 Nef (100%). Internalization function was assessed by flow cytometry following transfection of CEM T cells as described in the Methods. **(B)** The relative abilities of representative Nef clones from subtype A (red), subtype C (green) and subtype D (blue) and their respective mutants at positions 8 and 11 to internalize SERINC3 are displayed. **(C)** The relative abilities of a representative participant-derived subtype B Nef clone and its respective reversion mutations at positions 8 and 11 to internalize SERINC3 are shown. **(D)** Amino acid frequencies at Nef codons 8 and 11, based on 3775 Nef sequences representing HIV-1 subtypes A, B, C and D obtained from the Los Alamos National Labs HIV sequence database, are shown. **(E)** The relative infectivities of NL4.3-derived viruses encoding S8R or I11G mutations produced in the presence of SERINC3 are shown. Statistical analyses were conducted using the unpaired Students T test. Significant differences compared to Nef controls are indicated by asterisks (* = p<0.05; ** = p<0.01; *** = p<0.001).

Our codon-function analyses indicated that polymorphisms at Nef residues 8 and 11 should attenuate its ability to internalize SERINC3, but not SERINC5. To confirm this observation and to assess the impact of these polymorphisms on two of Nef’s other major functions, namely CD4 and HLA class I internalization, we introduced S8R and I11G substitutions into NL4.3 Nef. These alternative amino acids at residues 8 and 11 are common in our dataset and are predicted to selectively impair SERINC3 internalization. Both mutants displayed normal abilities to internalize CD4, HLA class I and SERINC5, but SERINC3 internalization was severely reduced (50% activity for S8R; 0% activity for I11G) (Fig 4A). To confirm that this loss of function was not particular to HIV subtype B, we introduced S8R or I/V11G substitutions into selected subtype A, C and D Nef clones that displayed normal abilities to internalize SERINC3. SERINC3 internalization was reduced in all cases, though to varying extents (Fig 4B). Since many circulating subtype B Nef clones encode both R8 and G11 polymorphisms and display impaired SERINC3 internalization, we attempted to rescue this activity in a subtype B Nef clone with relatively poor function by reverting these amino acids. While individual reversions of R8S or G11V showed no effect in this clone, the double-reversion (R8S and G11V) restored SERINC3 internalization function from 45 to 79%, relative to NL4.3 (Fig 4C). These results confirm that polymorphisms at Nef residues 8 and 11 can impair its ability to internalize SERINC3. Furthermore, this impairment appears to be selective, since no major effects were observed on Nef’s abilities to internalize CD4, HLA class I or SERINC5.

To further examine global Nef sequence diversity at residues 8 and 11, we calculated amino acid frequencies at these sites based on 3775 publicly available Nef sequences from subtypes A, B, C or D (www.hiv.lanl.gov/; query restricted to one Nef sequence per individual). Consistent with our panel of 339 Nef clones, Serine and Valine were highly conserved at positions 8 and 11, respectively, in subtype A, C and D sequences, but these residues were highly polymorphic in subtype B (Fig 4D). This is consistent with our observation that circulating subtype A, C and D strains are more likely to retain SERINC3 antagonism activity compared to subtype B strains.

Finally, to investigate the effect of polymorphisms at Nef residues 8 or 11 on viral infectivity, we introduced S8R or I11G mutations into NL4.3, generated viruses in the presence of SERINC3, and measured their infectivity, as described previously. Overall, viruses encoding S8R or I11G were 3.3-fold and 4.4-fold less infectious compared to NL4.3, respectively (Fig 4E). These results indicate that both mutations impaired Nef’s ability to counteract SERINC3 to a degree that was similar to that of a Nef-deleted virus (which displayed a ~4-fold reduction in this assay).

### Nef residues 8 and 11 contribute to co-localization with SERINC3

**Fig 5:**
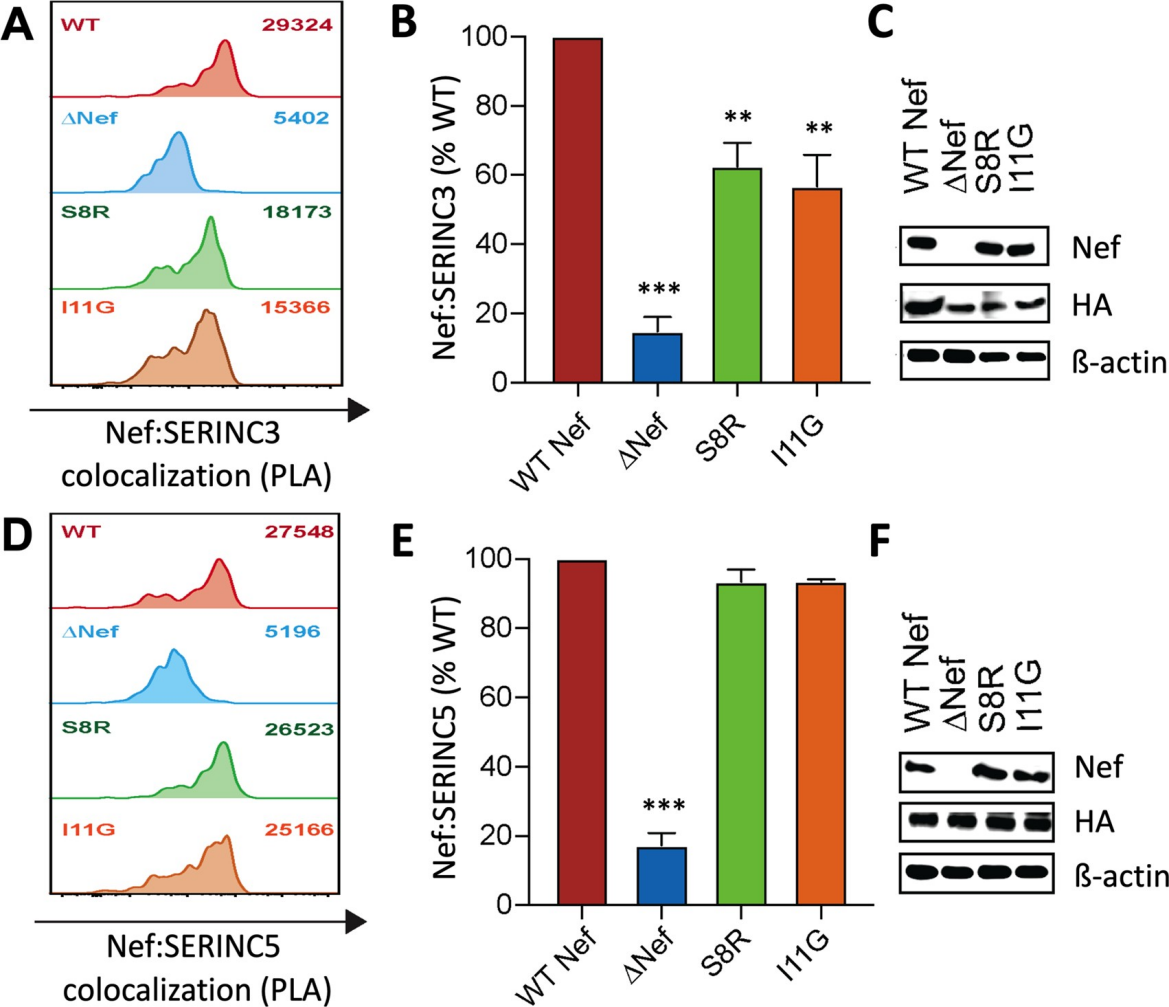
Polymorphisms at Nef residues 8 and 11 selectively impair SERINC3 co-localization. Flow cytometry-based proximity ligase assays (PLA) were used to detect co-localization of Nef with SERINC3(iHA) or SERINC5(iHA), as described in the **Methods**. (**A**) Histograms display PLA signal (median fluorescence intensity, MFI; upper right) for CEM T cells co-transfected with SERINC3(iHA) and either WT Nef (NL4.3), empty vector (ΔNef), Nef S8R or Nef I11G mutants. **(B)** Normalized PLA values for Nef and SERINC3 co-localization, relative to WT Nef, are shown. **(C)** Steady-state protein expression is shown for Nef (top) and SERINC3(iHA) (middle), determined by Western blot using the same anti-Nef and anti-HA antibodies as used in PLA. Cellular β-actin was included as a loading control (bottom). The results for similar studies using SERINC5(iHA) are shown, with representative PLA histograms (**D**), normalized PLA values (**E**) and protein expression (**F**) displayed. Results shown in (**A**) and (**D**) are representative of data collected in at least three independent experiments, each done in triplicate. Statistical analyses (unpaired Students T test) were conducted using median values from independent experiments. Significant differences compared to WT Nef are indicated by asterisks (** = p<0.01; *** = p<0.001).

Since polymorphisms at Nef residues 8 and 11 negatively impacted SERINC3 internalization but had negligible effects on CD4, HLA class I or SERINC5 internalization, we hypothesized that these residues are crucial for Nef to interact with SERINC3. To explore this possibility, we used the proximity ligase assay (PLA) to investigate the intracellular co-localization of Nef with SERINC3(iHA) and SERINC5(iHA), as described in Methods. This method generates a fluorescent signal if two proteins of interest are located within ~40 nm of each other inside the cell [39], thus allowing potential interactions to be measured semi-quantitatively as the median fluorescence intensity (MFI) of cells by flow cytometry. Using PLA, we observed a ~7-fold induction of fluorescent signal in cells expressing wild type Nef and SERINC3 (Fig 5A) or SERINC5 (Fig 5D) compared to background signal generated by control cells lacking Nef. In contrast, the MFI of cells expressing Nef S8R or I11G mutants and SERINC3 was markedly lower (62% or 55% relative to wild type Nef, respectively) (Fig 5B), suggesting that the impaired internalization function of these mutants is due at least in part to a reduced ability to co-localize with SERINC3. Notably, the same mutants displayed PLA signals similar to wild type Nef in the presence of SERINC5 (both >95%) (Fig 5D and 5E), which also consistent with their ability to internalize SERINC5 as efficiently as wild type Nef. Finally, similar detection of steady-state Nef and SERINC(iHA) by Western blot (Fig 5C and 5F) indicated that variation in PLA signal was unlikely to be due to differences in protein expression or antibody recognition.

## Discussion

HIV group M subtypes A, B, C and D collectively account for ~75% of the global pandemic. Nef is a crucial determinant of viral pathogenesis, and also ranks among the most variable regions in the HIV genome [40, 41], suggesting that differences in Nef function may contribute to clinical outcome. Our analysis of 339 diverse Nef clones from these four major HIV subtypes demonstrates that subtype A and C clones displayed lower ability to internalize SERINC5 compared to those from subtypes B and D. This raises the intriguing possibility that an attenuated capacity to counteract SERINC5 may contribute to decreased pathogenesis in the context of subtype A or C infection, which has been seen in regions where these strains co-circulate with subtype D [24]. Also of note, subtype D Nef clones were most efficient at counteracting both SERINC3 and SERINC5, suggesting that this feature could also be linked to the increased pathogenesis of this subtype. Indeed, over half (43 of 85, 51%) of subtype D clones displayed an ability to internalize both restriction factors at levels above the cohort-wide median for each function, while only 26% of subtype A, 23% of subtype B, and 35% of subtype C Nef clones met this threshold (S2 Fig).

In contrast to a prior study of SIV Nef isolates that reported a correlation between SERINC5 antagonism activity and prevalence of SIV strains in wild primates [27], our results indicate that Nef’s ability to counteract SERINC5 (or SERINC3) does not obviously correlate with global HIV subtype prevalence: indeed, subtype C accounts for nearly 50% of all HIV infections worldwide [21], yet subtype C Nef clones displayed lower median SERINC5 internalization function compared to those from subtypes B and D in our assays. This apparent discrepancy between SIV and HIV may be due to a greater contribution of population-level factors other than viral fitness to the spread of HIV subtypes globally, including founder events [42], human social changes and complex transport networks [43, 44].

Our finding that Nef-mediated antagonism of SERINC3 displayed a distinct functional hierarchy among viral subtypes compared to that of SERINC5 indicates that these two closely related Nef activities are in fact functionally separable and further suggests that *in vivo* selection pressures to maintain these two Nef activities might differ. Indeed, the majority of subtype B Nef clones internalized SERINC5 efficiently but were impaired in their ability to counteract SERINC3, whereas SERINC3 antagonism activity was maintained in most Nef clones derived from subtypes A, C and D. We also discovered that these differences in SERINC3 antagonism could largely be attributed to residues in Nef's N-terminal anchor region, which allows the protein to interact with the inner leaflet of the plasma membrane as well as a variety of cellular kinases that are critical to its function [45]. In particular, we demonstrated that polymorphisms at residues 8 and 11 reduced Nef’s ability to co-localize with SERINC3 (but not SERINC5) based on PLA staining. These results suggest that Nef engages SERINC3 and SERINC5 at different locations within the cell, an intriguing possibility that will require more study. Notably, we observed that subtype B strains displayed the poorest SERINC3 antagonism activity, on average, of all subtypes tested, which is consistent with the presence of R8 and G11 as consensus amino acids in these isolates. Though the implications of this for viral transmission and spread remain unknown, we note that HIV subtype B dominates the Western world as a result of founder effects [46], but it has not survived in central Africa where it originated [47], possibly because it was outcompeted by the diverse HIV strains circulating there.

While we have not explored the mechanisms that underlie differential Nef function among viral subtypes, it is conceivable that host evolutionary pressures have contributed at least in part. With respect to this, we acknowledge that the subtype A, C and D clones used in our study were collected primarily from female participants in East and Southern Africa who were likely infected via heterosexual sex, while subtype B isolates were collected primarily from Canadian participants, mostly men who have sex with men. Our study was not designed to evaluate the potential impact of sex and additional research on this topic is needed. SERINC expression levels can also vary among individuals [48], which may influence the effectiveness of Nef-mediated SERINC antagonism and also contribute to population-level selection of Nef alleles. Additional studies are warranted to examine the contribution of host genetics and other aspects of the immune response on the antiviral activity of SERINC restriction factors.

The importance of Nef-mediated SERINC antagonism for HIV pathogenesis remains to be elucidated. Our understanding of SERINC restriction mechanisms, and Nef’s role as a mediator of viral infectivity (apart from its ability to counteract SERINC), continues to evolve [49, 50]. While we observed strong correlations between Nef’s ability to internalize SERINC proteins and its ability to enhance viral infectivity, we did not directly measure SERINC incorporation into virions. Indeed, while most studies have observed an inverse association between the amount of SERINC incorporated into virions and their infectivity [7, 8, 17], supporting Nef-mediated sequestering of SERINC as a major strategy to counteract its antiviral activity, some reports suggest that Nef can antagonize SERINC without altering its levels in virion particles [51, 52]; thus, other known or unknown [50] Nef activities may contribute to our observations. In addition, Nef’s ability to counteract SERINC3 or SERINC5 is likely to depend in part on the relative expression levels of each protein [51, 53]. In our studies, we titrated SERINC proteins to enhance detection of Nef’s effects, but this may not fully recapitulate natural variation in SERINC expression seen among diverse human populations. Nef expression (as detected by Western blot) did not correlate with SERINC internalization function in our assays (S3 Fig), but Nef stability varies substantially among isolates and might contribute in some cases. Furthermore, HIV Env can modulate viral sensitivity to SERINC5 [19] and a recent study found that SERINC5 binds to an “open” conformation of the Env trimer that is induced by the cellular CD4 protein [54], the presence of which can differ on the surface of infected cells in a Nef-dependent manner [55]. However, the relative impact of Env compared to Nef to SERINC antagonism has not been examined carefully and potential functional interactions between *nef* and *env* have not yet been studied in detail.

In summary, our results reveal HIV subtype-specific variation in Nef’s ability to counteract SERINC3 and SERINC5 that mirror subtype-specific associations with HIV disease progression in global regions where multiple HIV strains co-circulate. We also identify naturally occurring Nef polymorphisms that are associated with differences in these functions. In particular, polymorphisms at residues 8 and 11 in Nef's N-terminal anchor domain that selectively impair SERINC3 antagonism activity were found in all HIV subtypes, but are consensus in subtype B, consistent with poor conservation of this function in subtype B clones. Our observations raise the intriguing hypothesis that Nef—and in particular Nef's SERINC antagonism activity—contributes to subtype-specific differences in HIV pathogenesis. Larger genotype/phenotype-based analyses should be conducted to assess the impact of Nef on HIV outcomes in diverse global populations.

## Methods

### Human subjects

No human subjects were enrolled for this study. Plasma specimens were obtained from antiretroviral naïve chronically HIV-infected individuals, as described previously [30]. Subtype A and subtype D isolates were derived from two Ugandan sites: the Adherence Monitoring Uganda (AMU) cohort from Kampala [56] and the Uganda AIDS Rural Treatment Outcomes (UARTO) cohort from Mbarara [57]. Subtype B isolates were derived from the HAART Observational Medical Evaluation and Research (HOMER) Cohort from British Columbia, Canada [58, 59]. Subtype C isolates were derived from the Sinikithemba cohort from Durban, South Africa [60]. Specimens were selected based on the availability of stored plasma or first-round plasma RNA-derived PCR products spanning the *nef* region [58].

### Ethics statement

This study was approved by the Research Ethics Boards at Simon Fraser University and the Providence Health Care/University of British Columbia. Human subject protocols were approved by REBs at the Mbarara University of Science and Technology, University of Kwa-Zulu Natal, or Providence Health Care/University of British Columbia. Participants provided written informed consent or historic specimens were anonymized according to REB-approved procedures for secondary use.

### Cell lines

CEM-A*02 cells were derived from CEM (a female human acute lymphoblastic leukemia CD4 T cell line) by stably transduction of HLA-A*02:01 using a retroviral vector (murine stem cell virus; Clontech), selected using puromycin, and maintained in RPMI-1640 media supplemented with 10% FBS, 2 mM L-glutamine, 1000 U/mL Penicillin and 1 mg/mL Streptomycin (all from Sigma-Aldrich) at 37°C with 5% CO_2_. HEK293T cells (a female human embryonic kidney cell line) and TZM-bl reporter cells (a female human carcinoma cell line, derived from Hela) were cultured in DMEM supplemented with 10% FBS, 2 mM L-glutamine, 1000 U/mL Penicillin and 1 mg/mL Streptomycin.

### Reagents

The following materials were obtained through the NIH AIDS Reagent Program, Division of AIDS, NIAID, NIH: HIV-1 NL4.3 infectious molecular clone (pNL4.3), from Dr. Malcolm Martin [61]; and TZM-bl cells, from Dr. John C. Kappes and Dr. Xiaoyun Wu [62–66]. Rabbit polyclonal anti-Nef antiserum was obtained from the NIBSC Center for AIDS Reagents program (cat. # ARP444, from Dr M Harris). Mouse anti-HA.11 antibody was purchased from Biolegend (clone 16B12) and rabbit polyclonal anti-SERINC3 antiserum was purchased from Abcam (cat # ab65218). pX330-U6-Chimeric_BB-CBh-hSpCas9, used for gene editing of *SERINC3*, was obtained from Addgene (plasmid #42230, gift from Dr. Feng Zhang) [67, 68].

### Generation of plasmids and expression constructs

Single phylogenetically representative Nef clones from 360 participants were isolated and characterized previously [30]. Briefly, patient-derived HIV *nef* alleles were amplified from plasma using nested RT-PCR and cloned into pSELECT-GFPzeo expression plasmid (Invivogen). For this study, Nef clones that displayed poor steady-state detection by Western blot were excluded, resulting in a total of 339 clones (92 subtype A, 91 subtype B, 71 subtype C, and 85 subtype D). These *nef* sequences are available as Genbank accession numbers KC906733 –KC907077.

A SERINC5 variant encoding an internal HA tag (iHA) was sub-cloned from pBJ5-SERINC5(iHA) [8] into a pSELECT plasmid lacking GFP (p-SELECT-ΔGFP). An analogous SERINC3(iHA) construct was synthesized as a gBlock Gene Fragment (Integrated DNA Technologies; Accession Number NM_006811) with an internal HA tag inserted between amino acids 311 and 312, a Kozak sequence (GCCGCCACC) inserted upstream of the start codon, and unique restriction enzyme cut sites BamHI and SacII located at the 5’ and 3’ ends, respectively, which was subsequently cloned into pSELECT-ΔGFP.

### Site-directed mutagenesis

Point mutations in reference Nef strains and primary Nef clones were introduced using overlap PCR extension, as previously described [69].

### SERINC, CD4 and HLA class I internalization assays

To assess Nef-mediated internalization of SERINC3 or SERINC5 from the cell surface, 1 × 10^6^ CEM-A*02 CD4 T cells were co-transfected with 1 μg of pSELECT-GFPzeo encoding *nef* and 5 μg of pSELECT-*SERINC3(iHA)*-ΔGFP or pSELECT-*SERINC5(iHA)*-ΔGFP by electroporation in 150 μL OPTI-mem medium (Thermo Fisher Scientific) using a BioRad GenePulser MXCell instrument (square wave protocol: 250 V, 2000 μF, infinite Ω, 25 millisecond single pulse). Cultures were recovered for 20–22 hours with 350 μl of R10+ medium (RPMI-1640 supplemented with 2 mM L-glutamine, 1000 U/ml Penicillin and 1 mg/ml Streptomycin, all from Sigma-Aldrich) at 37°C plus 5% CO_2_. Following this, 2.5 × 10^5^ cells were stained with 0.5 μg of Alexa Fluor 647 anti-HA.11 (BioLegend) and analyzed by flow cytometry for GFP expression (marker for transfected cells) and cell surface SERINC expression (HA tag stain) using a Millipore Guava 8HT instrument. The median fluorescence intensity (MFI) values of SERINC3/5 for each Nef clone were normalized to the positive (pSELECT-*nef*_WT (NL4.3)_-GFPzeo) and negative (pSELECT-Δ*nef*) controls using the formula: (MFI_ΔNef_−MFI_CLONE_)/(MFI_ΔNef_−MFI_WT_) × 100, such that Nef function less than or greater than wild type Nef is represented by values of <100% or >100%, respectively. Nef-mediated internalization of CD4 and HLA class I was assessed by flow cytometry using a similar co-transfection protocol, as described previously [30, 33].

### Generation of SERINC3 knockout HEK293T cell line

We generated a SERINC3 knockout (KO) derivative of the HEK293T cell line using CRISPR/Cas9 technology. Parental 293T cells were transfected with a pX330-based plasmid encoding previously described target sequences [7] using DNAfectin 2100 (Applied Biological Materials) and then serially diluted into 96-wells to isolate clonal progeny. Disruption of *SERINC3* in 293T clones was confirmed by Western blot using rabbit polyclonal anti-SERINC3 (Abcam).

### Viral infectivity assays

To assess viral infectivity, 8 × 10^5^ SERINC3 KO 293T cells were seeded on 6 well plates and transfected with 2 μg pNL4.3ΔNef, 30 ng pSELECT-*SERINC3(iHA)*-ΔGFP or pSELECT-*SERINC5(iHA)*-ΔGFP and 10 ng pSELECT-n*ef*-GFP using DNAfectin 2100 (Applied Biological Materials). Culture supernatants containing viruses were harvested at 48-hours post-transfection, centrifuged for 10 minutes at 1000 x g to pellet residual cells or debris and then stored as aliquots at -80°C until use. Viral quantification was done using p24 ELISA (XpressBio). A standardized amount of each virus (5 pg or 10 pg p24) was used to infect 1 x 10^4^ TZM-bl reporter cells in a 96-well flat bottom plate in triplicate. Infectivity was measured at 48 hours using Steady-Glo Luciferase (Promega) on a Tecan Infinite M200 PRO plate reader.

### Proximity Ligase Assay (PLA)

PLA was conducted using Duolink flowPLA Detection Kit—Far Red Mouse/Rabbit kit (Millipore-Sigma). Briefly, 1 x 10^6^ CEM A*02 cells were transfected with 1 μg of pSELECT-Nef-GFP and 5 μg pSELECT-*SERINC3/5(iHA)*-ΔGFP as described above. After 20 hours, cells were treated with BD Cytofix-Cytoperm solution (BD Biosciences), and co-stained with rabbit polyclonal anti-Nef (cat. # ARP444; NIBSC Center for AIDS Reagents) and mouse anti-HA.11 (Biolegend, clone 16B12) overnight at 4°C. Secondary incubation anti-rabbit PLUS and anti-mouse MINUS probes, ligation, amplification and wash steps were completed in solution as directed by the manufacturer. The median fluorescence intensity (MFI) of cells was quantified on a GUAVA 8HT flow cytometer (Millipore) and results were normalized to those of parental NL4.3 Nef (100%) and empty vector (0%).

### Western blot

Steady-state Nef expression levels were assessed by transfecting 2.5 x 10^6^ CEM-A*02 CD4 T cells with 10 μg of pSELECT-*nef*-GFPzeo via electroporation as described above. After 24 hours, cells were pelleted, lysed and prepared as described previously [31]. Nef was labeled using a polycolonal rabbit serum (cat. # ARP444; NIBSC Center for AIDS Reagents) (1:2000) followed by staining with HRP Donkey anti-rabbit IgG antibody (BioLegend) (1:5000). To validate CRISPR/Cas9-mediated knockout of SERINC3 in 293T cells, 2 x 10^6^ parental or KO cells were pelleted and lysed. Endogenous SERINC3 expression was detected using rabbit polyclonal anti-SERINC3 antibody (Abcam ab65218) (1:1000 dilution) followed by staining with HRP Donkey anti-rabbit IgG antibody (BioLegend). Proteins were detected using Clarity Western ECL substrate (Bio-rad) and visualized on ImageQuant LAS 4000 imager (GE healthcare).

### Statistical analysis

Nef sequences were aligned to the reference strain HXB2 (GenBank accession number K03055) and insertions with respect to HXB2 were removed using an in-house alignment algorithm based on the HyPhy platform [70]. Nef polymorphisms associated with differential *in vitro* SERINC3 or SERINC5 internalization function within each subtype were identified using a custom Perl script. For every Nef polymorphism present in 3 or more unique HXB-aligned sequences per subtype (i.e. a frequency of 3.3–4.2%), clones were repeatedly grouped according to the presence versus absence of the observed variant and the nonparametric Mann-Whitney U-test was then used to compare median SERINC downregulation function between groups. Multiple comparisons were addressed using q-values, the p-value analogue of the false discovery rate (FDR). FDR is the expected proportion of false positives among results deemed significant at a given p-value threshold (e.g. at a q ≤ 0.1, we expect 10% of identified associations to be false positives). Analysis of population level HIV diversity at Nef residues 8 and 11 was done using the AnalyzeAlign tool provided on the HIV Sequence Database at the Los Alamos National Laboratory (www.hiv.lanl.gov). For this, the “Web alignment” dataset was selected, which has been curated to contain only a single sequence per participant and to remove low quality sequences.

All other statistical analyses were performed using Prism v.8 (Graphpad). Since functional data were not distributed normally (based on the Kolmogorov-Smirnov test), non-parametric tests were employed. The Mann-Whitney U test was used to compare differences in median Nef function between subtypes. The Spearman rank sum test was used to examine correlations between Nef functions. Results of two-tailed tests were considered significant if the p-value was less than 0.05.

## Supporting information

S1 Fig Assay to measure SERINC3 and SERINC5 internalization functions of HIV Nef. (TIF)

S2 Fig Associations between SERINC3 and SERINC5 internalization functions among HIV Nef clones. (TIF)

S3 Fig Associations between SERINC, CD4 and HLA class I internalization functions and protein expression among HIV Nef clones. (TIF)

S1 Table Nef polymorphisms associated with SERINC5 internalization function (all p<0.05). (XLSX)

S2 Table Nef polymorphisms associated with SERINC3 internalization function (all p<0.05). (XLSX)

S3 Table Nef polymorphisms associated with both SERINC3 and SERINC5 internalization function (all p<0.05). (XLSX)
