# Supplementary figures and images for: Variation in HIV-1 Nef function within and among viral subtypes reveals genetically separable antagonism of SERINC3 and SERINC5
Source: PLoS Pathog. 2020 Sep 14;16(9):e1008813. doi: 10.1371/journal.ppat.1008813 (PMC7515180; doi:10.1371/journal.ppat.1008813)

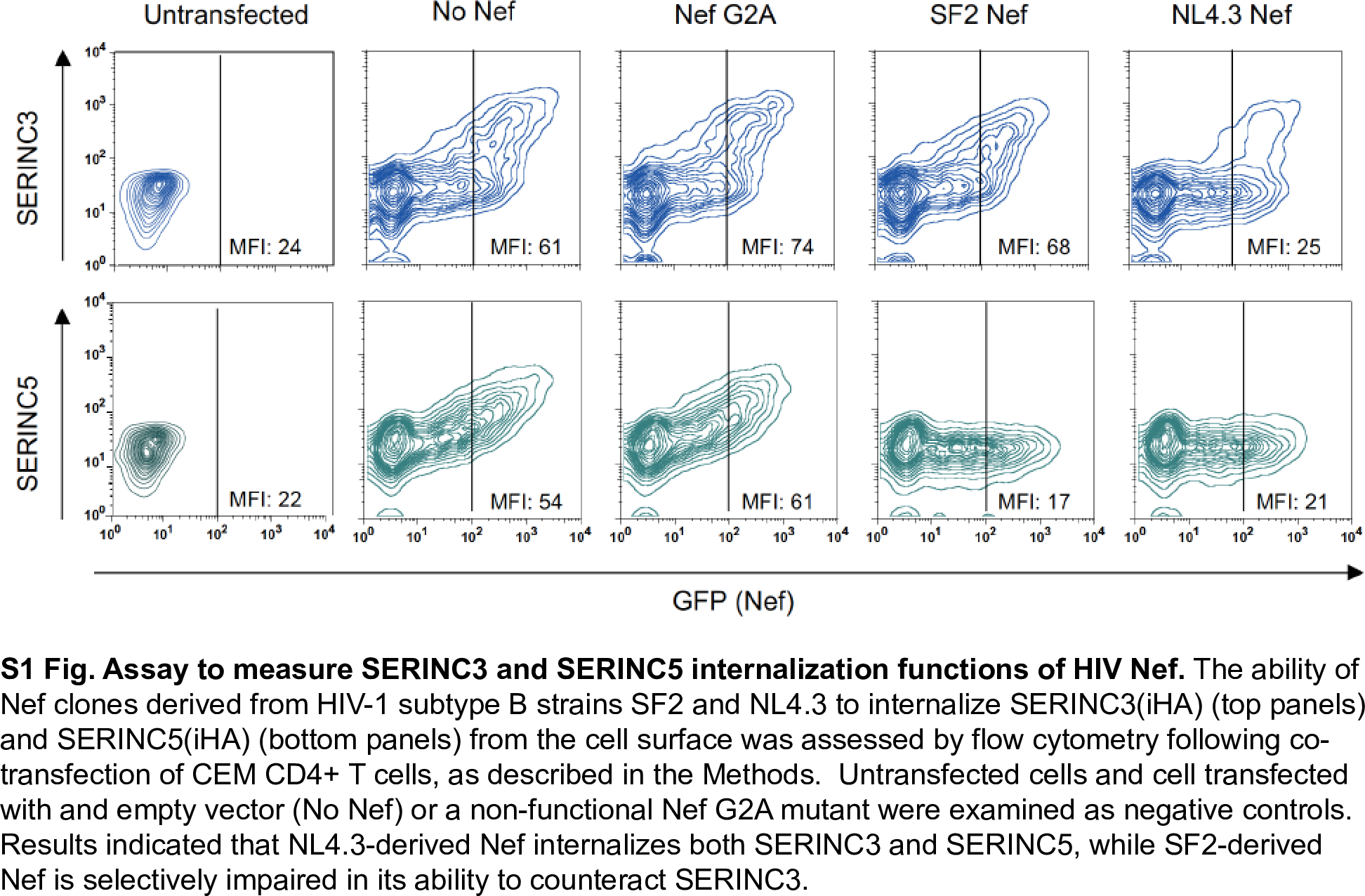

Supplement: S1 Fig — (TIF) [file ppat.1008813.s001.tif]

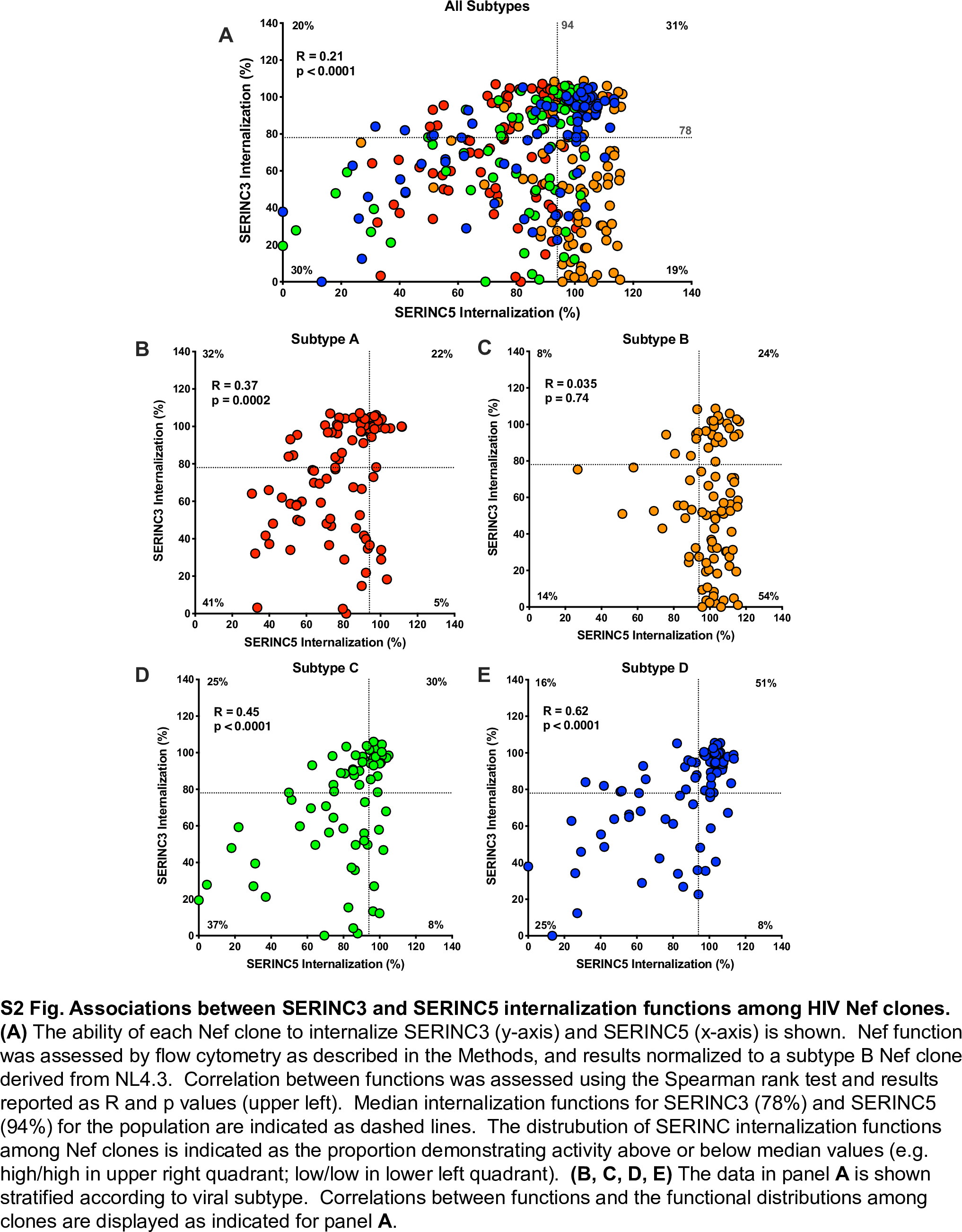

Supplement: S2 Fig — (TIF) [file ppat.1008813.s002.tif]

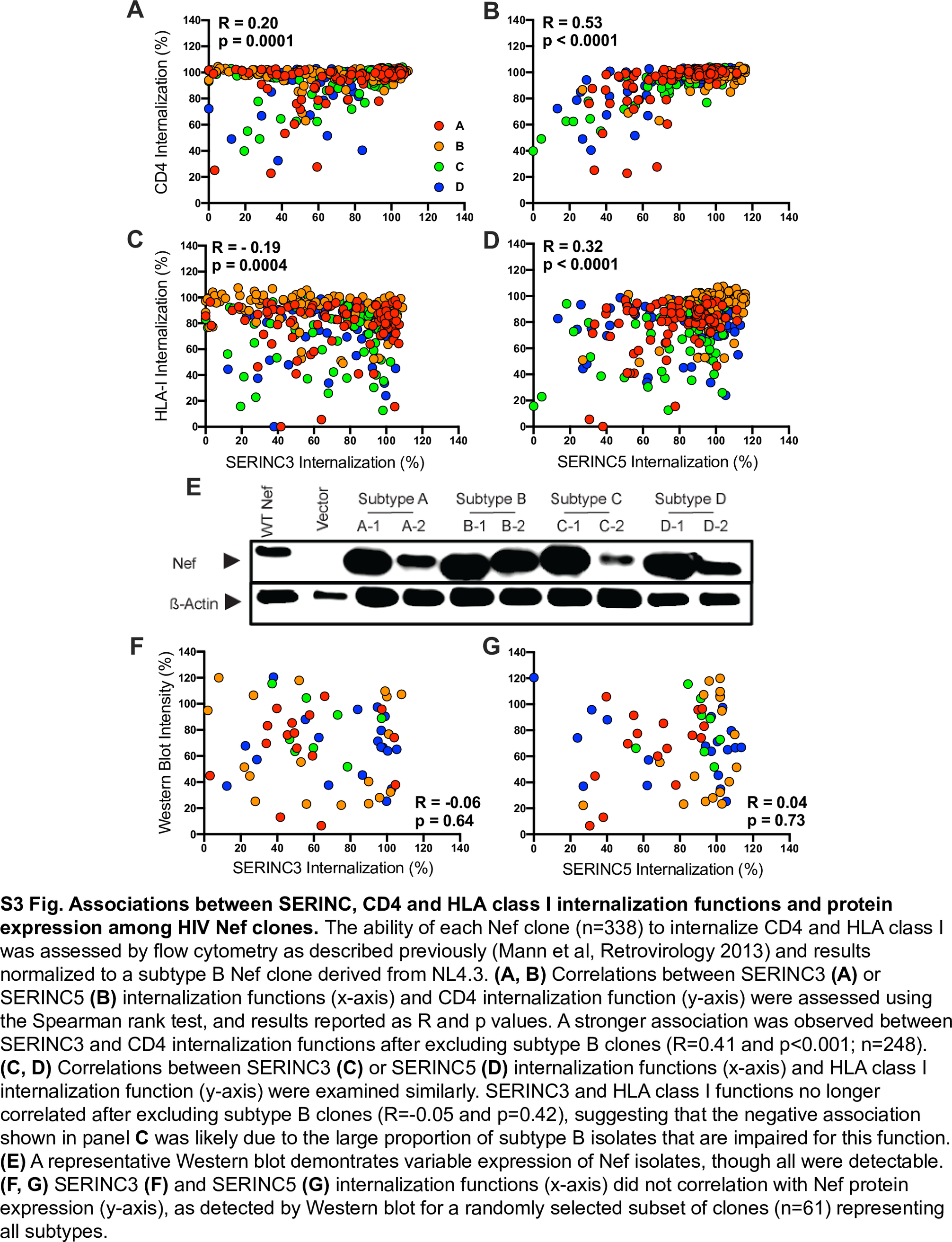

Supplement: S3 Fig — (TIF) [file ppat.1008813.s003.tif]
